# Expected Impacts of Connected Multimodal Imaging in Precision Oncology

**DOI:** 10.3389/fphar.2016.00451

**Published:** 2016-11-29

**Authors:** Marco D. Dominietto, Enrico Capobianco

**Affiliations:** ^1^Department of Biomedical Engineering, Biomaterials Science Center, University of BaselAllschwil, Switzerland; ^2^Center for Computational Science, University of MiamiMiami, FL, USA

**Keywords:** physiological maps, tumor heterogeneity, multimodal imaging, complex networks, digital biomarkers, multicentric clinical trials

“Insanity is doing the same thing over and over again and expecting a different result.”

Albert Einstein

## Precision oncology: genetic therapy and standard treatments

Precision oncology refers to a systematic assessment of cancer, from genomics to translational applications. Its relevance depends particularly on evidences of tumor heterogeneity: no two cancers are the same, and often in two subjects can behave very differently. We tend to consider heterogeneity manifested at the clinical level, but indeed it starts at the genetic level. Roughly speaking, cancer arises in most of the cases from a group of mutated genes, in particular altered oncogenes and tumor suppressors that cooperate to promote tumor progression. Driven by the altered genes, the pathological behavior of tumor tissue follows. Because for the same tumor the oncogenes can significantly vary from patient to patient, an ideal assessment of genomic alterations at an individual basis will turn to the development of *ad hoc* genetic therapies removing the primary cause of the disease. However, the implementation of genetic therapies is still far from representing clinical routine.

Common therapies to treat solid tumors consider symptoms and are aimed at blocking the disease progression by killing any single cancer cell. They consist of a combination of (a) Surgical resection of the neoplastic mass, followed by (b) Radiation therapy to sterilize the surgical bed and tumor border, and (c) Systemic chemotherapy, if necessary. Thus, on one hand a local control of the disease is needed, but with chemotherapy on the other end the goal becomes to prevent the dissemination of malignant cells into other organs, and consequently avoid the formation of metastases. Each combination of these three modalities ultimately tries to avoid any possibility of replication. In particular, the target of radiation treatment is the cellular DNA that can be broken by means of direct or indirect radiation effects (Lomax et al., 2013). Instead, chemotherapy targets specific processes underlying the regulation of tumor growth through biomarkers that identify tumor cells. In both therapeutic contexts, the biological rationale hypothesizes higher toxicity to malignant than to normal cells.

Two weaknesses emerge from this approach: (a) The differences between tumor and healthy cells may be not so significant to avoid damage to healthy tissue and (b) The heterogeneity of tumor phenotypes is not allowing development of absolute target-specific drugs. This is the case for example for a class of anti-angiogenic drugs that target the vascular endothelial growth factor (VEGF) with the aim to avoid the formation and development of new vessels. Recently, it has been demonstrated that VEGF is expressed only in smaller vessel and not in the more mature and larger vessels (Nagy and Dvorak, 2012). Therefore, this kind of drug affects only partially the tumor vascular network, reducing the overall effectiveness.

## Physiological maps: deciphering complexity

The idea of identifying common biomarkers, or a restricted group of them shared between all types of tumors, has inspired oncological research since the beginning. While this goal has not been achieved, research in this direction has highlighted two main features of tumor behavior: heterogeneity and uncertainty.

Heterogeneity may find in some cases a multitude of causes beyond clonal evolution or environmental differences, generating functional diversity influencing response to therapy and prognosis, and determining uncertainty with regards to both biological and technological factors (Magee et al., 2012). Overall, the two features have translated into complexity which has drastically limited the discovery impact of efficient drugs.

Advances toward deciphering complexity have involved quantification by modern diagnostic imaging technique routinely used in clinics, say Magnetic Resonance Imaging, Positron Emission Tomography, Computer tomography, and Ultrasound. These allow the acquisition of many features related with tumor development, and suggest progress in quantitative imaging (Mountz et al., 2014). The metabolic activity of tumors can be mapped following glucose, oxygen, and free fatty acids consumptions, which constitute the major source of energy that cells need for replication. In turn, we can have a complete evaluation of the vascular network architecture, physiology, and its hierarchical connections with the healthy counterpart. Furthermore, it is possible to determine the state of the tumor microenvironment, which is responsible for tissue invasion and metastasis formation. A great innovation of modern diagnostics is the possibility to monitor dynamic physiological process in 3D-mode with a resolution reaching microscopic structure. This means that it has become possible to determine not only the average stage of the tumor, but to draw a detailed map of its stages at a microscopic scale.

**Figure 1:**
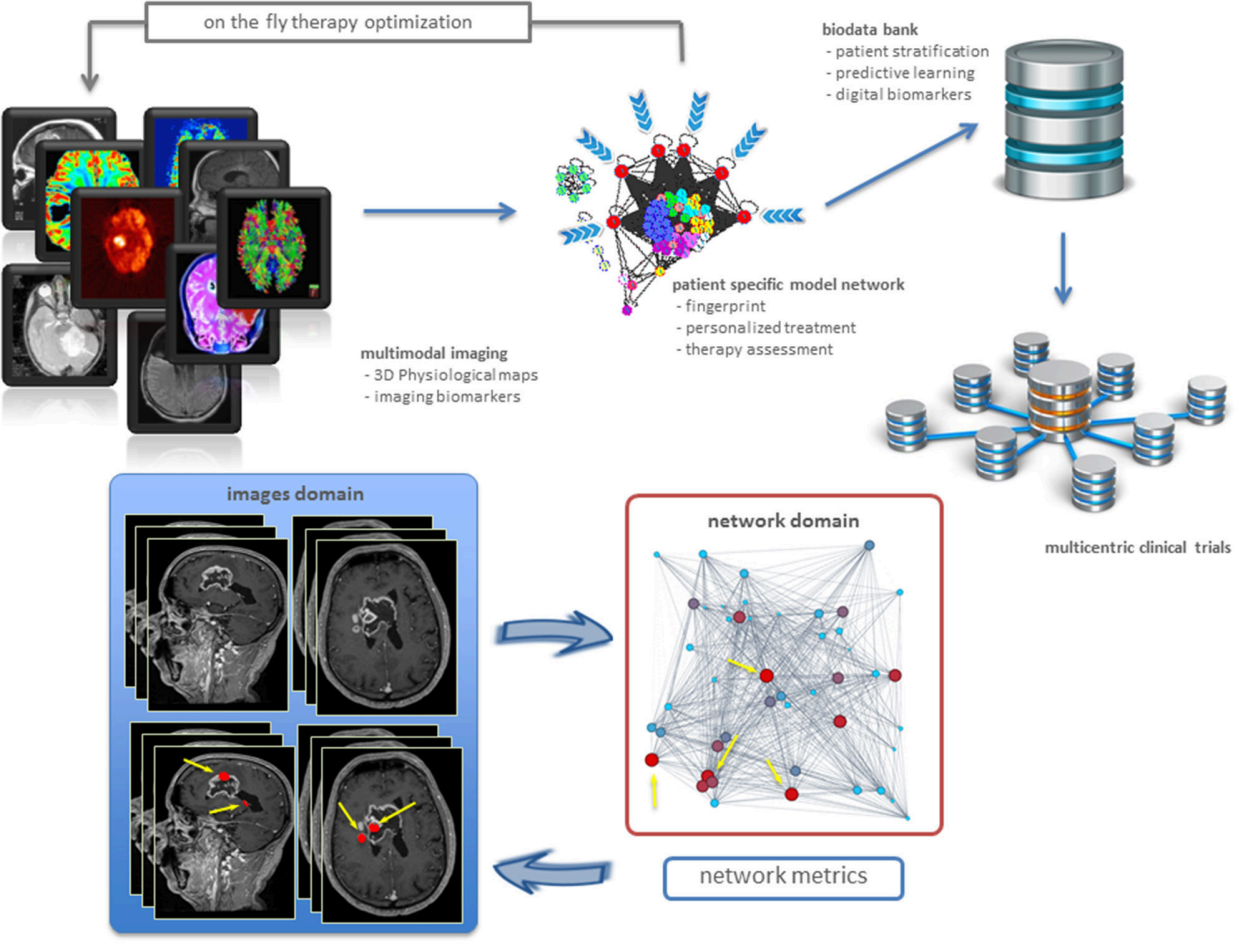
**Precision oncology advances**. From imaging biomarkers to network quantifications and modeling, toward new patient stratifications and clinical trials **(top)**. Example of a clinical application in a patient with brain tumor **(bottom)**. Sagittal and axial MRI images showed the presence of highly heterogenic glioblastoma. Metrics readout performed in the network domain has hierarchically classified proliferative regions through a gradient, from red spots corresponding to high proliferation rate, to light blue identifying low proliferation rate. As a result, we have mapped four most proliferating regions with red nodes in the network from the MRI image voxels (indicated by yellow arrows). Not only this establishes inter-domain correspondence, but also emphasizes additional red nodes as supplemental network interactions, useful for predictive purposes.

Physiological maps are the key to explore complexity, by inspecting the interactions occurring inside and outside the cancer tissue. Intuitively, the tumor is seen as a group of microscopic elements organized in macroscopic structures interacting together and with the host organ. Therefore, we can model the tumor as a complex network where the nodes are the microscopic elements that are connected together on the basis of their physiological behavior (Dominietto et al., 2015). Nodes that behave similarly form macroscopic structures, or clusters, in turn interacting between them (intra-cluster), and with the surrounded healthy tissue. Figure 1 offers a general and personalized view of the novel approach. The bottom panel establishes a correspondence between image and network domains through markers of proliferative regions.

“…In the era of internet, the real knowledge does not come from the amount of information we are able to process/store, but from the number of connections we are able to establish among them.”

Somewhere in the web

## Biomarkers, imaging biomarkers, digital biomarkers: role in precision oncology

Biomarkers represent a fundamental aspect of cancer research, and are used to detect cancer or direct cancer therapy. However, both the molecular nature and the functional architecture of cancer are incredibly sophisticated and coordinated, revealing interactions with immune system response, microenvironment, metabolism, life style, nutrition, etc., which translate into a myriad of complex signals hard to be accounted for. Similarly, this high complexity is reflected into the need for multimodal imaging combining a variety of features bringing additional prediction power to molecular and functional measures. This data complementarity paves the way for new quantifications and models, mapping information from cells to voxels in a multiscale inference approach ranging from macroscopic scale (morphological features) to mesoscopic scale (cellular level) and finer scales (imaging to investigate metabolism, stress response, adaptation etc.; Yankeelov et al., 2013). Imaging biomarkers build robust measures and lead clinical decisions. Being non-invasive, imaging allows repeated measurements over time thus capturing patient-, tissue-, or tumor specific heterogeneity and inspecting the microenvironment under both stationary and non-stationary (perturbed) conditions (Farwell et al., 2015). This explains why molecular imaging has a relevant role as a predictive biomarker and as an early indicator of response to therapy (Mankoff et al., 2014).

Networks allow in principle to develop patient specific tumor models based on the measured pathophysiological maps (Dominietto and Capobianco, 2016). While many network metrics may be in principle established to extract information about the cancer system under study, most networks explain relationships between bioentities by a few standard metrics (Roy, 2014). The specific metric underlying the topology and dynamics of such networks can be then used for two distinct aims in cancer research. First, to identify the critical nodes that when targeted by a drug, may cause the collapse of the network (catastrophic effects). This may explain cancer dynamics linked to disruption of metastatic and resistance mechanisms, etc. Second, to build digital biomarkers constituting the fingerprints of specific tumors. In this case, one must identify a variety of cancer features which call for measurement and integration. In particular, it is crucial to indicate how drugs contribute to such next generation of markers. Figure 1 (bottom) shows an example of digital biomarkers, indicating high proliferative regions subject to mapping between image and network domains. These markers highlighted as red nodes were determined by means of the metrics performed on the network, and also directly on the medical image to prioritize delivery treatments as ionizing radiation and drugs to such locations.

## Impacts on targeted cancer therapy

Personalized cancer therapy is strictly associated with precision oncology, in turn highly dependent on biomarkers. Among these, imaging biomarkers are destined to be central due to several unique properties, especially the ability to examine *in vivo* the tumor microenvironment in perturbed or unperturbed state, including of course early and follow-up stages of response to therapy. This step is to be developed further in terms of quantification of measurements, validation of data acquisition, statistical methods for image quality and reliability of evidences, and standardization of digital records. Enabling newly designed qualitative and quantitative protocols, both for the detection and classification of tumor type and stage, will make substantial impacts on clinical decision making regarding patients selection. Clinical imaging is thus expected to be central in future patient-specific cancer therapies and for drug efficacy assessment (Gatenby et al., 2013).

Looking forward, new patient stratifications based on increased and diverse biomarkers (biomarkers varieties) will have a dramatic impact in the design and development of clinical trials. Among the next generation of biomarkers, digital ones refer to physio-pathological and behavioral conditions that are not considered in standard or traditional ways, depending on new medical devices, apps from mobile technologies, etc. The role of digital biomarkers is to elucidate significance of factors such as lifestyle, nutrition, wellness, environment, etc. such that they can be distinguished from confounders.

The correct classification of the disease and the personalized treatment will restrict the variability emerging from subject heterogeneity. Patients with a specific tumor will be cured with specific treatment. Such aspect will drastically improve the robustness of multicentric trials that usually suffer of non-homogeneity issues. Moreover, the aim of therapy follow-up will not be confined to the simple macroscopic determination of tumor geometry as volume and mass indicators. The new biomarkers will offer the possibility to evaluate the regression of the disease at a pathophysiological level, allowing the modification of the therapy “on the fly” and the calibration of the dose preventing unnecessary side effects. This is a key aspect as currently most clinical trials offer a randomized approach delivering snapshots of treatments effectiveness, on average. Instead, optimization for progressive and recurring diseases requires a sequential clinical decision approach, over time and space, both dimensions being useful to monitor changes in patient's profile, i.e., biomarker characteristics, genetic profiles and medical histories, and response to treatment.

## Big data

In healthcare, images, biological/genetic data, clinical records, results of genetic test and other diagnosis, and biometric profiles are increasingly generated and need to be stored as electronic health records (EHRs) whose volume, variety and velocity are known challenges. Scalable big data tools need integration with analytical methods to enable decision support clinical systems to become routine and (i) Improve disease management; (ii) Harmonize validations of medical treatments and responses to therapies; (iii) Focus on risk prediction and prevention. The bottleneck is clearly the interoperability that can be achieved when facing heterogeneity and diversity of data, in turn affecting their complete accessibility and usability for scopes of knowledge discovery and prediction.

## Concluding remarks

In this Opinion, we have expressed a few key points, summarized below:

- Medical imaging is already a key component in personalized medicine, but quantification and representation improvements are needed for superior precision.
- Molecular biomarkers will be supported by imaging and digital biomarkers, with impact on decisions referred to choice of treatment and assessment of response to it.
- Prevention relying on newly conceived screening programs will be more effective due to the better monitoring and prediction induced by digital medicine.
- Clinical trials will become more dynamically optimized by the means of clinical decision support systems.

Precision Oncology is what we have discussed and much more. Currently, the field is undergoing a major change also due to the switch from microarrays to high-throughput sequencing, which has allowed a wider and in-depth analysis of genomes, transcriptomes, epigenomes, etc. Imaging has remained a bit lateral to such developments, despite the tremendous impact of technology and the relevance especially in clinical diagnostics and therapy assessment. Looking at the genetic layers of information in an integrated way and predictively with imaging, will increase our understanding of phenotypic alterations, and influence both response to therapy and prognosis.

## Author contributions

Both authors have equally contributed to the ideation and the preparation of the manuscript.

### Conflict of interest statement

The authors declare that the research was conducted in the absence of any commercial or financial relationships that could be construed as a potential conflict of interest.
